# Closed-incision negative pressure therapy as a strategy to reduce sternal wound infection in identified high-risk patients: a multicentre propensity matched study

**DOI:** 10.1093/icvts/ivae056

**Published:** 2024-04-08

**Authors:** Rona Lee Suelo-Calanao, Andrea D’Alessio, Sandra Hutton, George Krasopoulos, Vijayakumar Muppiri, Carly Cartwright, Ahmed Parvez, Nicolas Nikolaidis, Mahmoud Loubani

**Affiliations:** Castle Hill Hospital, Hull University Teaching Hospital, Hull, United Kingdom; Oxford University Hospital, Oxford, United Kingdom; Oxford University Hospital, Oxford, United Kingdom; Oxford University Hospital, Oxford, United Kingdom; The Royal Wolverhampton NHS Trust, Wolverhampton, United Kingdom; The Royal Wolverhampton NHS Trust, Wolverhampton, United Kingdom; The Royal Wolverhampton NHS Trust, Wolverhampton, United Kingdom; The Royal Wolverhampton NHS Trust, Wolverhampton, United Kingdom; Castle Hill Hospital, Hull University Teaching Hospital, Hull, United Kingdom

**Keywords:** Sternal wound infection, Closed incision negative pressure therapy, Sternotomy

## Abstract

**OBJECTIVES:**

The premise of this retrospective study was to evaluate the intraoperative use of closed-incision negative pressure therapy (ciNPT) to help reduce the incidence of postoperative sternal wound infections in multimorbid patients with an elevated risk of developing a sternal wound infection post cardiac surgery versus a cohort that received standard-of-care dressings.

**METHODS:**

Data for all adult patients were collected from each cardiothoracic surgery unit across 3 hospitals in the United Kingdom. High-risk patients had 2 or more recognized risk factors. Fisher’s exact test (two-tailed) and unpaired *t*-test were used to help analyse categorical and continuous data. Propensity matching was performed to compare the 2 groups.

**RESULTS:**

A total of 5,288 patients who had cardiac surgery were included. Propensity matching led to 766 matched cases. There were significantly fewer sternal wound infections in the ciNPT group [43 (5.6%) vs 119 (15.5%) cases; *P* = 0.0001], as well as fewer deep sternal wound infections [14 (1.8%) vs 31 (4.0%) cases; *P* = 0.0149] and superficial sternal wound infections [29 (3.8%) vs 88 (11.4%) cases; *P* = 0.0001]. A higher mean length of stay in the ciNPT group was statistically significant (11.23 ± 13 vs 9.66 ± 10 days; *P* = 0.0083) as was a significantly higher mean logistic European System for Cardiac Operative Risk Evaluation (EuroSCORE) (11.143 ± 13 vs 8.094 ± 11; *P* = 0.0001). A statistically significant higher readmission to the intensive care unit due to sternal wound infection was noted for the controls [16 (2.08%) vs 3 (0.39%) readmissions; *P* = 0.0042].

**CONCLUSIONS:**

The ciNPT appears to be an effective intervention to help reduce the incidence of sternal wound infection in high-risk individuals undergoing cardiac surgery.

## Graphical Abstract

**Figure:**
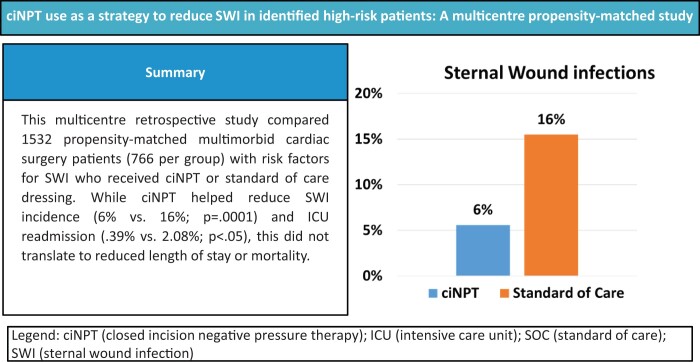

## INTRODUCTION

The development of a sternal wound infection (SWI) represents a major complication following cardiac surgery [1]. Aside from potential life-threatening consequences, SWI is associated with a prolonged length of stay (LOS) in the hospital and, according to the European Health Service, may add an estimated 19 billion Euros of additional health care costs [2]. It has been previously demonstrated that avoiding virulent bacterial contamination that can lead to mediastinitis and the degradation of sutured skin, especially in high-risk patients, is integral to helping prevent postoperative complications associated with SWI [3, 4]. This postoperative complication is most prevalent in patients with a medical history of obesity, diabetes, chronic obstructive pulmonary disease (COPD) and advanced age [5].

According to Friberg *et al.*, reported cases of SWI immediately postoperatively are approximately 1%–2%; however, the rate increases to 5%–8% and a further 6%–10% after 2 and 3 months, respectively [6]. The National Institute of Health and Care Excellence (NICE) has noted that a lack of a postoperative follow-up at 1 month may also be characterized as a risk factor and has recommended closed-incision negative pressure therapy (ciNPT) use as an intervention for cardiothoracic surgery patients determined to be at high risk of developing an SWI [7]. The ciNPT is applied as an incision-management dressing that can administer -125 mmHg for up to 7 days. The mechanism of action of ciNPT resides in its ability to help hold incisional edges together and to help bolster and stabilize closed incisions [8]. The ciNPT is also enlisted to remove fluid and infectious materials and to help present a barrier against external contaminants [9].

Preventing SWI is important in reducing patients’ mortality and morbidity and the cost of care to putatively provide substantial savings in any surgical service by reducing the length of hospital stay [10]. Our goal was to evaluate the impact of administering ciNPT immediately postoperatively to cardiothoracic patients identified as high-risk as part of an incision management strategy at 3 cardiac surgery units in the United Kingdom (UK) to help reduce the incidence of SWI.

## MATERIALS AND METHODS

### Ethics statement

The study was approved by the Hull University Teaching Hospitals NHS Trust Clinical Audit and Governance Board as a multicentre clinical audit (approval number NA.2021.091) granted on 30November 2021. It was also approved by the clinical audit departments of all participating hospitals. Patient informed consent was not obtained due to the anonymity of all data.

### Study design

This is a multicentre, retrospective study to identify any putative clinical benefit of ciNPT use as a perioperative strategy in helping to reduce SWI incidence in patients considered to be at an elevated risk of developing said surgical site complications. With the approval of the institutional review boards of 3 health-care institutions, de-identified clinical data were collected from the patient databases of 3 hospitals and provided to the lead investigator for analysis by the respective cardiothoracic surgery units that participated in the study.

### Setting

The 3 units that utilized ciNPT (3M Prevena Incision Management System, 3M Company, St Paul, MN, USA) postoperatively for incision management were invited to participate in the multicentre audit. Upon agreement, completed audit applications were submitted by each cardiothoracic surgery unit to their respective institutional review board. Upon approval, independent data gathering was performed by each unit using the audit criteria as a guide (Table 1) as well as an adapted scheme for characterizing wound classification, depth and description (Table 2). De-identified data were sent to the lead investigator for consolidation and analysis. Consent was not required because no cardiac surgery patients were contacted for information.

**Table 1:** Audit criteria

Age
BMI
COPD
Diabetes mellitus
Type of operation
Logistic EuroSCORE
Postoperative sternotomy wound infection
Wound classification*
Treatment for wound infection
Antibiotics
Debridement
Rewiring
Negative pressure wound therapy dressing
Plastic surgical reconstruction
Length of postoperative hospital stay
Readmission to the ICU and subsequent hospital stay due to wound infection
Death

* Sternal wounds severity are classified using Table 2.

**Table 2.** Wound classification based on anatomical site plus (adapted from Suelo-Calanao et al. 2020)*

Classification	Depth	Description
Type 1a	Superficial	Skin and subcutaneous tissue
Type 1 b	Superficial	Exposure of sutured deep fascia
Type 2a	Deep	Bone exposure, sternum with stable steel suture
Type 2 b	Deep	Bone exposure, sternum with unstable steel suture
Type 3a	Deep	Necrotic bone exposure, or fractured, unstable sternum, exposed heart
Type 3 b	Deep	Type 2 or 3 with septicemia

### Study patients/patient selection

**Figure 1:**
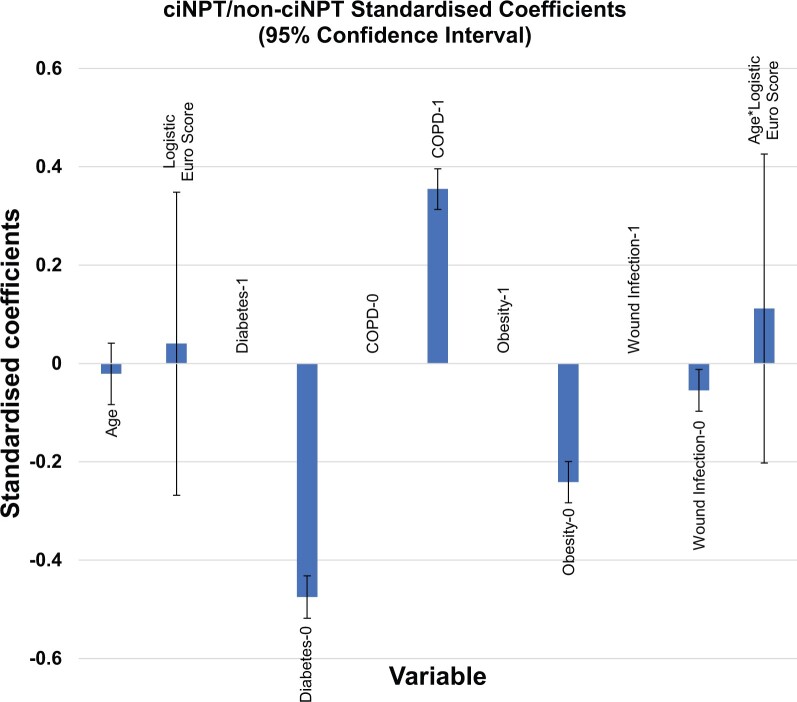
Standardized coefficients of the logistic EuroSCORE and 4 risk factors for sternal wound infection development. ciNPT: closed-incision negative pressure therapy; COPD: chronic obstructive pulmonary disease.

The study included all adult patients who underwent cardiac surgery via median sternotomy between January 2013 and December 2021 from either of 3 cardiothoracic surgery centres in the UK: Castle Hill—Hull University Teaching Hospital, Oxford University Hospital, and The Royal Wolverhampton NHS Trust. Patients were identified as having received ciNPT (ciNPT group, *n* = 1060) or a standard dressing (control group, *n* = 4228) (Table 3). Patients were propensity matched using the following risk factors identified from our previous study (Ariyaratnam et al.): advanced age (above 80 years), diabetes mellitus, COPD and obesity [body mass index (BMI) > 30 kg/m^2^) as independent variables (Table 4 and Fig.1).

**Table 3:** Patient demographics, perioperative risk factors and type of surgery

	Control group (n = 4228)	ciNPT group (n = 1060)	*P*-value
Risk factors			
Age, mean (SD)	68.366 (10)	70.324 (8.47)	0.0001
Male	3181 (75%)	810 (76.4%)	0.0014
Female	1047 (24.7%)	250 (23.5%)	0.5605
Obesity (BMI >30 kg/m^2^)	1378 (5.65)	536 (4.07)	0.0001
COPD	510 (12.06%)	365 (34.4%)	0.0001
Diabetes	841 (19.8%)	588 (55.4%)	0.0001
Logistic EuroSCORE mean (SD)	8.094 (11.047)	11.143 (13.42)	0.0001
Type of Surgery			
CABG only	1586 (37.5%)	509 (48%)	0.0001
CABG + other	1076 (25.4%)	195 (18.3%)	0.0001
Valve only	690 (16.3%)	192 (18.1%)	0.0012
Valve + other	618 (14.6%)	77 (7.3%)	0.0001
Other cardiac operation	258 (6.10%)	87 (8.2%)	0.0005

BMI: body mass index; CABG: coronary artery bypass grafting; ciNPT: closed-incision negative pressure therapy; COPD: chronic obstructive pulmonary disease; EuroSCORE: European System for Cardiac Operative Risk Evaluation; SD: standard deviation.

**Table 4A-F.** Propensity matching data†

A. Summary statistics: closed-incision negative pressure therapy/non-closed-incision negative pressure therapy				
Variables	Categories	Counts	Frequencies	Percentage (%)
ciNPT	1	951	951	21.614
Non-ciNPT	0	3449	3449	78.386

B. Summary statistics: quantitative data							
Variables	Observations	Observations with missing data	Observations without missing data	Minimum	Maximum	Mean	SD
Age	4400	0	4400	17.000	90.000	69.435	9.280
Logistic EuroSCORE	4400	0	4400	0.800	95.270	8.405	10.880

C. Summary statistics: qualitative data				
Variables	Categories	Counts	Frequencies	Percentage (%)
Gender	0	3310	3310	75.227
	1	1090	1090	24.773
Diabetes	1	1185	1185	26.932
	0	3215	3215	73.068
COPD	0	3620	3620	82.273
	1	780	780	17.727
Obesity	1	995	995	22.614
	0	3405	3405	77.386
Wound infection	1	158	158	3.591
	0	4242	4242	96.409

D. Correspondence between categories of the response variable and the probabilities: closed-incision negative pressure therapy/non-closed-incision negative pressure therapy	
Categories	Probabilities
0	0
1	1

E. Standardized coefficients: closed-incision negative pressure/non-closed-incision negative pressure						
Source	Value	Standard error	Wald χ^2^	Pr > χ^2^	Wald lower bound	Wald upper bound
Age	−0.021	0.032	0.439	0.508	−0.083	0.041
Logistic EuroSCORE	0.040	0.157	0.065	0.798	−0.268	0.348
Diabetes-1	0.000	0.000				
Diabetes-0	−0.475	0.022	466.968	< 0.0001	−0.518	−0.432
COPD-0	0.000	0.000				
COPD-1	0.355	0.021	281.583	< 0.0001	0.313	0.396
Obesity-1	0.000	0.000				
Obesity-0	−0.241	0.021	127.104	< 0.0001	−0.283	−0.199
Wound Infection-1	0.000	0.000				
Wound Infection-0	−0.055	0.022	6.370	0.012	−0.097	−0.012
Age*Logistic	0.112	0.160	0.487	0.485	−0.202	0.426

F. Summary of the matched observations					
Categories	Number	Matched	Percentage (%)	Unmatched	Percentage (%)
1	951	766	81%	185	19%
0	3449	766	22%	2683	78%

ciNPT: closed-incision negative pressure therapy/non-closed-incision negative pressure therapy.

EuroSCORE: European System for Cardiac Operative Risk Evaluation.

COPD: chronic obstructive pulmonary disease.

COPD: chronic obstructive pulmonary disease; EuroSCORE: European System for Cardiac Operative Risk Evaluation.

Cost: 2.565

†Interactions/level: 2; matching method: optimal algorithm Euclidean distance; number of matches: 1:1; caliper: 0.10. The measure and parameters used by the software to identify a 1:1 matched ratio between the 2 groups.

* Sigma; confidence interval (%): 95; tolerance: 0.001; number of removed observations: 888. 80 years old and above.

### Variables of interest

The variables of interest for this investigation were cardiothoracic surgery type and patients with risk factors for developing an SWI who underwent cardiac surgery. The following cardiothoracic surgical procedures were performed via a median sternotomy: isolated coronary artery bypass graft (CABG), isolated heart valve repair/replacement, CABG with other surgery, heart valve repair/replacement with other surgery and other cardiac procedures. Patients with 2 or more of the following risk factors—obesity, advanced age, COPD and diabetes mellitus—were noted. Other variables such as sex, LOS and types of wound infections were also documented. To quantify the preoperative mortality risk profile, the logistic European System for Cardiac Operative Risk Evaluation (EuroSCORE) was also calculated for patients within both cohorts (Table 3).

### Data sources and study size

Patients with BMI > 30 kg/m^2^, 80 years old and above, a Spirometry test for COPD and blood glucose level for diabetes were noted, and those who had 2 or more of these conditions were considered high risk. A propensity matching was performed using only the stated conditions as covariants, which led to the size of the sample.

### Bias

A 1:1 matching was performed to obtain a treatment group and a control group with similar covariates. Because the study had both continuous and nominal data, a lower caliper was used to lessen the chance of bias.

### Statistical methods/analysis

The propensity matching was performed using the XLSTAT 2022.4.1.1366 software (Lumivero, Denver, CO, USA) [11]. This study primarily applied propensity score matching to 2 treatment cohorts rather than to various groupings, given the existence of some crucial unresolved issues (e.g. assessing balance among baseline variables, matching distance and caliper width choice) [12].

In this study, BMI, age, COPD and diabetes were used as covariates. Patients were matched 1:1 with an optimal algorithm Euclidean distance using a 0.1 caliper, with a confidence interval (CI) of 95% and 0.001 tolerance. Propensity score matching resulted in 766 patients matched in both the control and the ciNPT groups.

According to the Monte Carlo simulations, using a caliper width up to a maximum of 0.2 would putatively disregard approximately 98% of the bias in the crude estimator, thereby generating CIs that would be on the order of the correct coverage rates when at least 1 of the covariates is continuous data [12].

Whereas categorical data were conveyed as percentages, all continuous data (e.g. patient age, logistic EuroSCORE, LOS and extended LOS) were stated as mean ± standard deviation (SD). An unpaired, two-tailed *t*-test was used to calculate the *P*-value for all continuous data. The Fisher exact test of independence (for a two-tailed test) was used to calculate the *P*-value for all categorical data such as BMI, COPD, diabetes, mortality, sex or number of infections. To calculate the *P*-value for all categorical data (e.g. BMI, COPD, diabetes, mortality, sex or number of infections), the Fisher exact test of independence (for a two-tailed test) was used. A *P*-value of < 0.05 was recognized as statistically significant between the groups.

## RESULTS

### Participants and descriptive data

The total number of initial study participants was 5,288; the ciNPT group had *n* = 1060 and the control group had *n* = 4228. All adult patients with complete data shown in Table 1 were included Patient demographics and risk factors between the control and the ciNPT groups were compared. Both groups had a similar average age, incidence of comorbidities (BMI, COPD and diabetes) and type of surgery performed (Table 3). Using 4 identified risk factors (age, BMI, COPD and diabetes), the groups were propensity matched, which resulted in a total of 1,532 patients (control group, *n* = 766; ciNPT group, *n* = 766) included in the study (Table 4 and Fig. 1). Both groups were also examined using the following variables: cardiac surgery type, logistic EuroSCORE, SWI cases, SWI classification, length of postoperative stay from day of surgery to discharge, ICU re-admission due to SWI and subsequent hospital stay due to SWI (Tables 3, 5, and 6).

**Table 5:** Distribution of sternal wound infections among the matched controls and the closed-incision negative pressure therapy group

	Control group (n = 766)	ciNPT group (n = 766)	*P*-value
Total patients with SWI	119 (15.5%)	43 (5.6%)	**0.0001**
Superficial SWI	88 (11.4%)	29 (3.7%)	**0.0001**
Deep SWI	31 (4.04%)	14 (1.8%)	**0.0149**
**SWI classification** *			
Type 1A	63 (8.2%)	20 (2.6%)	**0.0001**
Type 1B	25 (3.2%)	9 (1.17%)	**0.0084**
Type 2A	15 (1.9%)	10 (1.3%)	0.4206
Type 2B	6 (0.78%)	4 (0.52%)	0.7531
Type 3A	7 (0.91%)	0	0.0154
Type 3B	3 (0.39%)	0	0.2495
Treatment administered			
Antibiotics	119(15.5%)	43(5.6%)	**0.0001**
Debridement	19(2.5%)	7(0.91%)	**0.0278**
Rewiring	13(1.7%)	4(0.52%)	**0.0479**
NPWD	28(3.6%)	9(1.17%)	**0.0023**
Plastic surgical intervention	7(0.91%)	0	**0.0154**

NPWD: negative pressure wound dressing ; SWI: sternal wound infection .

* SWI classification is described in Table 1.

**Table 6.** Postoperative length of stay, readmission to the intensive care unit, extended length of stay and deaths

	Control group (n = 766)	ciNPT group (n = 766)	*P*-value
Length of post-operative stay (mean ± SD)	9.661 ± 10.31	11.238 ± 13.129	0.0083
Readmission to ICU due to SWI, n (%)	16(2.08%)	3(0.39%)	0.0042
Subsequent hospital stay due to wound infection, days (mean ± SD)	256 days (16 ± 14)	36 (12 ± 5.35)	0.6358
Deaths	141(18.4%)	26(3.39%)	0.0001

ICU: intensive care unit; LOS: length of stay; SD: standard deviation; SWI: sternal wound infection.

A total of *n* = 109 (ciNPT) and *n* = 779 (control) patients were excluded in the matching due to incomplete data.

Compared to the ciNPT group, the control group contained a higher percentage of CABG + other (27.8% vs 8.9%, *P* = 0.0001) and valve + other cases (14.9% vs 5.8%, *P* = 0.0001) (Table 3). However, the ciNPT group had a higher percentage of cases for cardiac operations such as isolated CABG (48% vs 37.5%; *P* = 0.0001), valve repair/replacement alone (18.11 vs 16.3%; *P* = 0.0012) or other cardiac operations (8.2% vs 6.10%; *P* = 0.0005) (Table 3). For the control and ciNPT cohorts, the predicted operative mortality of patients was calculated using the logistic EuroSCORE model (Table 3). The mean logistic EuroSCORE captured statistical significance between cohorts (control group = 8.094 ± 11 vs ciNPT group = 11.143 ± 13; *P* = 0.0001) and helped to prevent the underestimation of individual patient risk to offer a more exact forecast where preoperative risk factors coexisted (Table 3).

#### Postoperative distribution of sternal wound infection between cohorts

Table 5 catalogues the distribution of SWI among patients receiving conventional dressings and ciNPT dressings. Of the 766 patients in the control group, a total of 119 (15.5%) patients had SWI. Of these, 88 patients had an SWI classified as a superficial infection and 31 were classified as deep sternal. A course of antibiotics treatment was administered to the 119 control group patients with SWI. Additionally, control group patients with SWI received the following interventions: debridement (*n* = 19), sternal rewiring (*n* = 13), application of conventional negative pressure wound therapy) (*n* = 28), and surgical revision by a plastic surgeon (*n* = 7).

Contrariwise, a total of 43 (5.6%) patients in the ciNPT group developed an SWI that was managed with a regimen of antibiotics. Within these patients, there were 29 SWIs characterized as superficial and 14 that were determined to be deep SWIs. Patients with SWI within the ciNPT group also had their wounds managed via debridement (*n* = 7), sternal rewiring (*n* = 4), and negative pressure wound therapy (*n* = 9); however, no patients were referred to plastic surgery for surgical revision. A lower incidence of SWI was noted among patients within the ciNPT group [*n* = 43 patients (5.6%)] relative to the control group [*n* = 119 patients (15.5%)], which was statistically significant (*P* = 0.0001). Additionally, patients within the ciNPT group also demonstrated a statistically significant lower incidence of deep SWI compared to the control group [*n* = 14 patients (1.8%) vs *n* = 31 (4.0%); *P* = 0.0149].

#### Length of inpatient hospital stay

Given its higher mean logistic EuroSCORE, the ciNPT group demonstrated a higher mortality risk profile evidenced by a longer mean LOS (11.2 ± 13.12 vs 9.6 ± 10.31 days; *P* = 0.0083), which was statistically significant (Table 6). Conversely, the control group demonstrated a higher rate of ICU readmission resultant of SWI (16 cases vs 3 cases, *P* = 0.0042), which was statistically significant. For patients with SWI who experienced a subsequent hospital stay due to wound infection, the total number of days for the control group was sevenfold higher than the ciNPT group (256 days vs 36 days) (Table 6). However, the mean hospital stay due to SWI was not statistically significant (16 ± 14 days vs 12 ± 5.35 days; *P* = 0.6358) (Table 6).

#### Cost comparison of surgical incision management modalities in patients with closed-incision negative pressure therapy

A simple cost analysis comparing the cost of treating control group patients who developed SWI and required readmission to the ICU against the cost of treating patients in the same category in the ciNPT group was performed. Sixteen patients in the control group, who were readmitted to the ICU due to SWI, had a total LOS of 256 days beyond the routine 5-day post-surgical LOS (Table 6). According to the data, the average cost of inpatient stay per day in the UK as of 2022 was £359 [3]. The extra expenditure for the control group totaled £91,904 (256 days x £359). However, the 3 patients who received ciNPT, who were readmitted to the ICU due to SWI, had a total LOS of 36 days beyond the routine 5-day postsurgical LOS (Table 7). The extra cost for the ciNPT group was £12,924 (36 days x £356). According to NICE, the cost associated with ciNPT use is £299 per unit, whereas the standard dressing costs £3.87 per unit [7]. The costs for managing the SWIs of the 16 control group patients and the 3 ciNPT group patients were £61.92 (£3.87 x 16 standard dressings) and £897 (£299 x 3 ciNPT units), respectively. Taken together, the total cost of an extended LOS post ICU readmission with incision management was £91,965.92 (£91,904 + £61.92) for the control group vs £13,821 (£12,924 + £897) for the ciNPT group. The £78,144.92 cost difference between cohorts would reflect a savings when ciNPT was utilized as a strategy to help aid in reducing the incidence of SWI (Table 7).

**Table 7.** Cost comparison between patients in the control group who developed sternal wound infections and received standard dressings and patients who received closed-incision negative pressure therapy

	Number of patients who developed SWI, n (%)	Readmission to ICU	Extended LOS after readmission to ICU	Cost/day/patients (NHS rate of £359)	Cost associated with sternal incision management (dressing)	TOTAL COST
Control group	119 (15.5%)	16(2.08%)	256 days	£91,904.00	£61.92	£91,965.92
ciNPT Group	43 (5.6%)	3(0.39%)	36 days	£12,356.00	£897	£13,821.00
					Difference of £78,144.92 as savings	

ciNPT: closed-incision negative pressure therapy; ICU: intensive care unit; LOS: length of stay; NHS: National Health Service; SWI: sternal wound infection.

## DISCUSSION

Evidence from various studies suggests that the use of ciNPT in adult cardiac surgery is a valid adjunctive therapy where the maximum benefit seems to be in helping to mitigate the incidence of deep SWI cases post sternotomy [1, 13, 14]. SWI is a grave postoperative complication associated with cardiac surgery, and recent data have indicated a 30% mortality rate among high-risk populations, with recent data reporting 30% associated mortality among high-risk populations [2]. Willy *et al.* noted obesity (BMI ≥ 30 kg/m^2^), diabetes mellitus, respiratory insufficiency and tobacco use as risk factors for the development of SWI [15]. A 2-centre randomized controlled trial previously reported that 1 independent predictive factor of an increased SWI rate is an elevated preoperative BMI in patients considered to be obese (BMI > 30 kg/m^2^) [16]. However, per a multivariate exact logistic regression model in Allen *et al.*, BMI was not a significant predictor for SWI or any sternal complication [17]. Our previous research in Ariyaratnam *et al.* identified 4 comorbidities (BMI, age, diabetes and COPD) that increase the risk of developing SWI [18].

In this study, the application of ciNPT to high-risk patients helped significantly to reduce the cases of SWIs (superficial and deep) and hospital LOS. A preliminary cost-of-care analysis suggests that ciNPT use may provide some putative cost saving in cardiac surgery relative to the standard-of-care dressing. Diabetes, COPD, advanced age and obesity represented the most common comorbidities in the study cohorts; however, patient demographics and comorbidities were similar between the control and the ciNPT cohorts because both groups were propensity matched. The current study also took into consideration a patient’s critical preoperative state by calculating the logistic EuroSCORE to assign a preoperative mortality risk grade. We reported statistical significance between the mean logistic EuroSCORE of the control cohort versus the ciNPT cohort. Additionally, certain surgical approaches may protract wound healing, which may then facilitate the occurrence of SSIs. For example, the use of internal thoracic arteries or mammary arteries in CABG operation combined with sternotomy has been reported as a potential operation-related risk factor for the development of SWIs [19, 20]. Because combined procedures (CABG + other, valve repair/replacement + other and other cardiac operation) were the most common cardiothoracic surgical interventions in our study, this may also be indicative of an elevated surgical risk factor for the development of SWI within our study population.

From a clinical research standpoint, SWI treatment post cardiac surgery is no longer sufficient because greater emphasis presently resides with SWI prevention as well as the health economics associated with managing this hospital-acquired infection [21]. Using a risk-stratification model, Fowler’s Score, Atkins *et al.* [10] cited diabetes and obesity as SWI risk factors. Colli and Camara [22] also used the same model, citing that ciNPT use was effective in preventing SWI complications up to the 30-day follow-up appointment. Grauhan *et al.* [23] assessed the effectiveness of ciNPT use on a larger group (*n* = 150) of obese patients. They reported that ciNPT use helped to significantly reduce the incidence of SWI in the obese population at 90-day follow-up appointments. A subsequent study by Grauhan *et al.* [24] assessed the effect of ciNPT utilization in the general population of post-sternotomy patients and confirmed their previous findings after 30 days follow-up. Our study appears to align with current evidence, because it demonstrated that high-risk patients receiving ciNPT exhibited a statistically significant lower incidence of SWI compared to the control group (5.6% vs 15.5%; *P* = 0.0001). Furthermore, 31 (4.0%) and 14 (1.8%) cases of deep SWI were noted in the control group and ciNPT group, respectively. The difference in these rates was also statistically significant (*P* = 0.0149) (Table 5). Our study reported that more control group patients required debridement (*n* = 19), sternal rewiring (*n* = 13) and intervention via plastic surgery (*n* = 7), whereas fewer ciNPT group patients had debridement (*n* = 7) or had sternal rewiring (*n* = 4) performed, and no plastic surgery referrals.

A mini review on complications of ciNPT by Sartipy et al. [25] and Li and Yu [26] raised potential safety concerns over its use. However, Dohmen *et al.* [27] produced cardiothoracic specific consensus recommendations on the use of ciNPT. Eleven cardiac and orthopaedic randomized controlled trials, retrospective studies and case studies were analysed. Taking into account previous studies, the authors were able to propose clear preoperative risk factors that predisposed patients to major infection after cardiac surgery. Both Dohmen *et al.* [27] and Biancari *et al.* [2] agreed that not all patients require ciNPT, but that the correct identification of those high-risk individuals was key in the provision of a cost-effective intervention. In our study, the mean LOS was higher in the ciNPT group (11.23 ± 13 days) compared to the control group (9.66 ± 10 days) (*P* = 0.0083), which is statistically significant. This result might be due to the greater representation of acute patients enrolled in the ciNPT group as evidenced by an elevated logistic EuroSCORE. On the other hand, the control group had significantly higher rates of readmission to the ICU due to SWI (3 vs 16 cases, *P* = 0.0042). Mean hospital stays post ICU readmission due to SWI were not statistically significant (16 ± 14 days vs 12 ± 5.35 days; *P* = 0.6358) despite the control group noting a sevenfold higher total number of days (256 days vs 36 days) (Table 6).

In the systematic review and meta-analysis by Biancari *et al.* [2], pooled rates were calculated from 10 included investigations and showed that 22.2 patients should be treated with ciNPT-based systems to help avoid a solitary SWI event. Although this result may seem to be a large number of patients, the putative benefit of ciNPT should be viewed in the context of an increased burden on resource utilization due to prolonged treatment of the complication. This is in addition to the significant clinical implication for the patient’s recovery, which could increase the risk of mortality.

Multiple reviews on the financial burden of treating SWI have been published [3, 28]. Recent data from the European Health Service noted an extra cost of 19 billion euros to treat SWIs [29]. Our simple cost analysis, which was based on the most current figures published by the King’s Fund [28], an organization that monitors key facts and figures about the UK National Health Service, determined that the average cost of hospitalization per day is £359. Given the extended total LOS for the control group (256 days) and the average cost of a simple surgical procedure (£2,817), the total expenditure for patients with SWI who require plastic surgery intervention is £111,623 (£91,904 + £19,719) (Table 7). Conversely, the cost associated with ciNPT use according to NICE and the total LOS of 36 days would amount to £13,821 (£897 + £12,924) (Table 7).

Admittedly, ciNPT may not be cost effective if used routinely when considering the cost of each device. Hawkins *et al.* [30] suggested that this treatment method may be cost effective only when used on patients at risk of developing deep SWI (1.3%), which could mean that fewer than half of identified high-risk cardiac patients may benefit clinically from the potential savings. However, there is no stratified scoring system available at present to measure the probability risk on the degree of SWI.

## LIMITATIONS

Our present study is limited by several considerations. First, despite the duration of the market availability of ciNPT, large, adequately powered clinical studies in the cardiac surgery sphere investigating ciNPT, which may help inform the surgeon or health care institution on the wider acceptance of this active wound care modality, are still wanting. Additionally, the high price point of ciNPT as an incision management approach relative to standard-of-care dressings, is still considered to be a cost prohibitive intervention in the absence of sufficient health economic evidence to justify its routine intraoperative administration during cardiac surgery to help reduce the incidence of SWI. Another possible limitation is that consideration was not afforded to microbiological characterization, postoperative low output syndrome, sternal stability and other perioperative concerns (cardiothoracic surgery type, procedure duration, urgency of the procedure, number of vessels reclaimed for total arterial revascularization) beyond multiple comorbid conditions that might have an influence upon the variance in SWI incidence between the cohorts. Our study is also limited by the rudimentary cost-comparison presented, where we cite a cost difference of £78,144.92 between members of the cohorts who developed SWI. Development of improved economic models designed to sensitively capture the quality-adjusted life years, risk stratification models, as well as studies incorporating health economic analysis, may assist in better defining cost offsets and economic end points to help mitigate the uncertainty of the clinical efficacy and cost-effectiveness of ciNPT in managing post-sternotomy incisions.

## CONCLUSION

The evidence presented in this multicentre retrospective study demonstrates that intraoperative ciNPT use should be viewed as an effective adjunctive intervention to help reduce SWI incidence in cardiac surgery patients with multiple comorbidities considered to have a high-risk of SWI development. Our study supports these findings, as demonstrated by the reduction of SWI cases in patients with an elevated risk for developing SWI. Although ciNPT use did not translate to a shorter hospital stay, it did reveal a lower incidence of readmission to the ICU and repeat surgical procedures that may render potential financial savings.

## Data Availability

Data are available and may be obtained from the corresponding author with the permission of ICVTS. The authors confirm that the data supporting the findings of this study are available within the article.

## Notes

This study was presented at the European Association for Cardiothoracic Surgery Meeting at the Austria Center Vienna, Vienna, Austria, 7 October, 2023 and at the Society of Cardiothoracic Surgery Annual Meeting, 21 March 2023, at the International Convention Centre in Birmingham, United Kingdom.

## ABBREVIATIONS

BMI: Body mass index
CABG: Coronary artery bypass graft
ciNPT: Closed-incision negative pressure therapy
COPD: Chronic obstructive pulmonary disease
EuroSCORE: European System for Cardiac Operative Risk Evaluation
LOS: Length of stay
NICE: National Institute of Health and Care Excellence
SWI: Sternal wound infections
UK: United Kingdom
