# Erratum to: Closed-incision negative pressure therapy as a strategy to reduce sternal wound infection in identified high-risk patients: a multicentre propensity matched study

**DOI:** 10.1093/icvts/ivae209

**Published:** 2024-12-21

This is a erratum to: Rona Lee Suelo-Calanao, Andrea D’Alessio, Sandra Hutton, George Krasopoulos, Vijayakumar Muppiri, Carly Cartwright, Ahmed Parvez, Nicolas Nikolaidis, Mahmoud Loubani, Closed-incision negative pressure therapy as a strategy to reduce sternal wound infection in identified high-risk patients: a multicentre propensity matched study, Interdisciplinary CardioVascular and Thoracic Surgery, Volume 38, Issue 5, May 2024, ivae056, https://doi.org/10.1093/icvts/ivae056

The originally published version of this article included supplementary material that did not pertain to the article. This material has been removed.

